# Inferring human history in East Asia from Y chromosomes

**DOI:** 10.1186/2041-2223-4-11

**Published:** 2013-06-03

**Authors:** Chuan-Chao Wang, Hui Li

**Affiliations:** 1 State Key Laboratory of Genetic Engineering and Ministry of Education Key Laboratory of Contemporary Anthropology, School of Life Sciences, Fudan University, 220 Handan Road, Shanghai, China

**Keywords:** East Asian populations, Y-chromosome, Migrations, Genetic structures

## Abstract

East Asia harbors substantial genetic, physical, cultural and linguistic diversity, but the detailed structures and interrelationships of those aspects remain enigmatic. This question has begun to be addressed by a rapid accumulation of molecular anthropological studies of the populations in and around East Asia, especially by Y chromosome studies. The current Y chromosome evidence suggests multiple early migrations of modern humans from Africa via Southeast Asia to East Asia. After the initial settlements, the northward migrations during the Paleolithic Age shaped the genetic structure in East Asia. Subsequently, recent admixtures between Central Asian immigrants and northern East Asians enlarged the genetic divergence between southern and northern East Asia populations. Cultural practices, such as languages, agriculture, military affairs and social prestige, also have impacts on the genetic patterns in East Asia. Furthermore, application of Y chromosome analyses in the family genealogy studies offers successful showcases of the utility of genetics in studying the ancient history.

## Introduction

East Asia is a sub-region of Asia, with a vast and diverse landscape. About 22% of the world’s population lives in East Asia, and more than 200 languages in seven linguistic families (Altaic, Austroasiatic, Austronesian, Daic, Hmong-Mien, Sino-Tibetan and Indo-European) are spoken in East Asia, which makes this region one of the world's most important places for studying human evolution, genetic diversity and interrelationships between genetics and cultures of human populations [1].

In the past few years, molecular anthropologists have made efforts to characterize the genetic diversity of East Asian populations by analyzing three types of genetic materials, that is, autosomal and X chromosomal DNA, the paternal Y chromosomal and maternal mitochondrial DNA (mtDNA). Autosomes and X chromosome are inherited from both the parents and are always jumbled by recombination. The non-recombining portion of the Y chromosome (NRY) is strictly inherited paternally and, therefore, is the best material to trace the paternal lineage of the populations with the additional advantages of small effective population size, low mutation rate, sufficient markers and population-specific haplotype distribution [2,3]. The debate on the single or multiple origins of anatomically modern humans has lasted for decades. In 1999, Su *et al.* used 19 stable and highly informative Y chromosome biallelic markers to assess the genetic structure of the paternal lineages in East Asia, and suggested that modern humans of African descent replaced the previous hominids living in East Asia [4]. In 2001, Ke *et al.* examined 12,127 male individuals from 163 populations using three Y chromosome biallelic markers (YAP, M89 and M130). They found that all the individuals carried a mutation at one of the three sites-YAP, M89 and M130 [5]. These three mutations (YAP+, M89T and M130T) coalesced into another mutation-M168T, which originated in Africa at around 44 thousand (95% confidence interval: 35 to 89 thousand years) years ago [3,5]. Although there have been possible gene flows between archaic hominids and modern humans [6-8], it is apparent that the majority of modern humans evolved recently in Africa; at least our Y chromosomes all came from Africa. The next question was how the early modern humans arrived in East Asia.

Climate has played an important role in human migrations, especially in the Last Glacial Period. The Last Glacial Period refers to the most recent glacial period from approximately 110 to 10 thousand years ago, covering the Paleolithic and Mesolithic periods of human history [9]. During this period, when the sea level was much lower than present, many of today's islands were joined to the continents, providing paths for modern human migrations. The maximum extent of glaciation (Last Glacial Maximum, LGM) was between 26.5 and 19 to 20 thousand years ago, when ice sheets were at their maximum extension and covered much of Asia, northern Europe and North America [10,11]. As a consequence, the living space for humans was probably very limited in the northern part of Asia. The ice sheets started to recede 15 thousand years ago and the temperature also began to rise. This period was a flourishing time for modern human migrations.

Here, we have focused on the migration histories of East Asian populations achieved by studying Y chromosome, and have discussed the patterns and microevolution during the initial human settlement and later migrations and expansions in East Asia. It is noteworthy at the very beginning that most time estimations mentioned in this review were achieved using Y chromosome short tandem repeats (STRs). Although this approach is correct in principle, there are still many ongoing debates about the best way to use STRs in haplogroup dating. In particular, there are two popularly used Y chromosome STR mutation rates, that is, the evolutionary rate [12,13] and the genealogical rate [14]. Choosing which mutation rate in Y chromosome dating is controversial, since the result can be almost three-fold difference. The high levels of homoplasy and varying mutational properties among loci also largely compromise the accuracy of estimation. Therefore, dates can only be intended as a rough guide for relative haplogroup ages.

## Review

### Northern route or southern route

Once it became generally accepted that modern humans evolved recently in Africa, the times and routes of migration to East Asia remained controversial. Three different models were insisted upon by different researchers. The first model postulated that northern populations of East Asia migrated to the south, and mixed with the Australian ancestors who had settled in Southeast Asia. The second model suggested that the northern populations of East Asia evolved from the southern settlers. However, a third model assumed that northern and southern East Asian populations evolved independently since the late Pleistocene more than 10,000 years ago [10,15,16].

**Figure 1:**
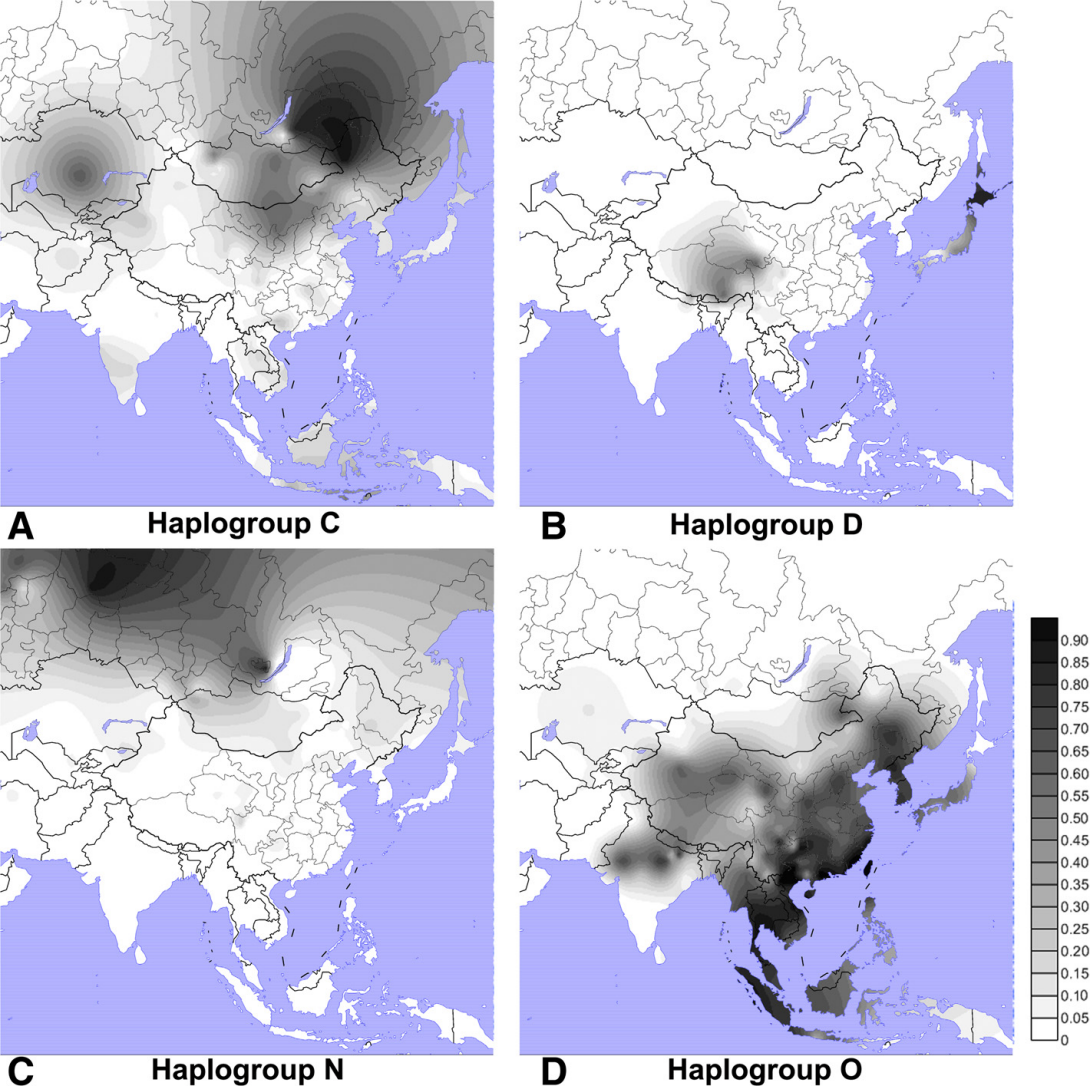
Geographic distributions of Y chromosome haplogroups C, D, N and O in East Asia.

There are four dominant Y chromosome macro-haplogroups in East Asia - O-M175, C-M130, D-M174 and N-M231 - accounting for about 93% of the East Asian Y chromosomes (Figure 1; phylogenetic trees showing every named marker are provided in Additional file 1). The other haplogroups, such as E-SRY4064, G-M201, H-M69I-M170, J-P209, L-M20, Q-M242, R-M207 and T-M70, comprise roughly 7% of the males in East Asia [15].

Haplogroup O-M175 is the largest haplogroup in East Asia, comprising roughly 75% of the Chinese and more than half of the Japanese population and, therefore, is associated with the major Neolithic migrants (Figure 1). O-M175 gave rise to three downstream haplogroups - O1a-M119, O2-M268 and O3-M122 - totaling 60% of the Y chromosomes among East Asian populations [17,18]. Haplogroup O1a-M119 is prevalent along the southeast coast of China, occurring at high frequencies in Daic speaking people and Taiwan aborigines [19]. O2-M268 accounts for about 5% of the Han Chinese [17]. O2a1-M95 is the most frequent subclade of O2, which is the major haplogroup in the Indo-China Peninsula, and is also found in many populations located in southern China and eastern India (such as Munda) [19,20]. Another subclade of O2, O2b-M176, is most frequent among Koreans and Japanese, and also occurs at very marginal frequencies in Vietnamese and Han Chinese [21,22]. O3-M122 is the most common haplogroup in China and is prevalent throughout East and Southeast Asia, comprising roughly 50 to 60% of the Han Chinese. O3a1c-002611, O3a2c1-M134 and O3a2c1a-M117 are the three main subclades of O3, each accounting for 12 to 17% of the Han Chinese. O3a2c1a-M117 also exhibits high frequencies in Tibeto-Burman populations. Another subclade, O3a2b-M7, reaches the highest frequency in Hmong-Mien and Mon-Khmer speaking populations, but accounts for less than 5% of Han Chinese [17,18].

**Figure 2:**
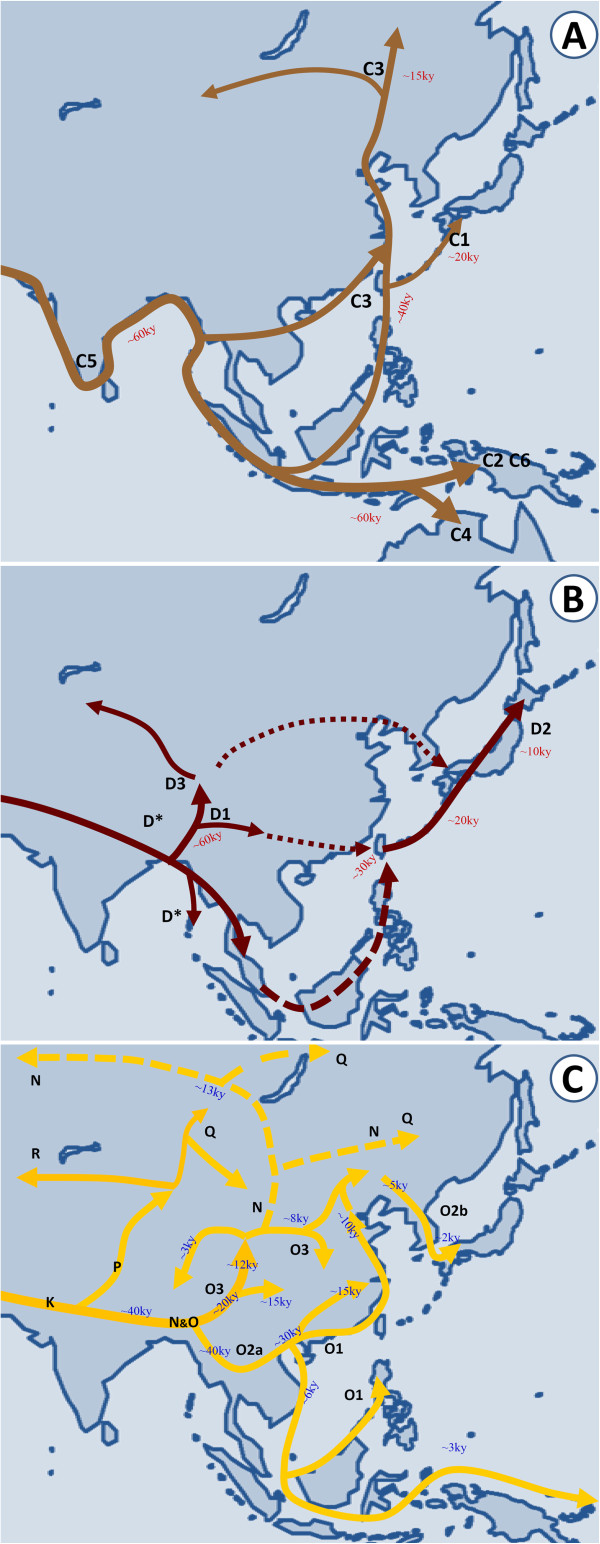
**Migration of the Y chromosome haplogroup C, D, N and O in East Asia.** Broken lines represent alternative migration routes.

Su *et al.* examined 19 Y-SNPs (including M119, M95 and M122) and three Y chromosome STRs in a large collection of population samples from a wide area of Asia. Principal component analysis of their study showed that all northern populations clustered together and were well included in the southern population cluster, and the southern populations were far more diversified than the northern populations. They concluded that the northern populations derived from the southern populations after the initial Palaeolithic peopling of East Asia. They also estimated the age of O3-M122 to be 18 to 60 thousand years using three Y-STRs under the single-step mutation model with a mutation rate of 0.18% per locus every 20 years, which might reflect the age of the bottleneck event leading to the initial settlement of East Asia [4]. In 2005, Shi *et al.*[18] presented a systematic sampling and genetic screening of haplogroup O3-M122 in more than 2,000 individuals from diverse populations in East Asia. Their data showed that the O3-M122 haplogroups in southern East Asia are more diverse than those in northern East Asia, supporting a southern origin of the O3-M122. The time of the early northward migration of O3-M122 lineages in East Asia was estimated about 25 to 30 thousand years ago using the average squared difference (ASD) method with an average Y-STR evolutionary mutation rate of 0.00069 per locus per 25 years [12,13]. Recently, Cai *et al.* examined the haplogroups O3a2b-M7 and O3a2c1a-M117 in Southeast Asian Mon-Khmer and Hmong-Mien speaking populations, and indicated a unidirectional diffusion through bottlenecks from Southeast Asia into East Asia about 19,000 years ago (also using the ASD method with an average Y-STR evolutionary mutation rate of 0.00069 per locus per 25 years) during the Last Glacial Maximum [23]. A general south-to-north Y-STR diversity decline was also observed in haplogroup O3a1c-002611, suggesting that haplogroup O3a1c also migrated northward along with other O3-M122 lineages [24]. Therefore, the southern route of the early human migration in East Asia, taking the largest Y haplogroups O, is supported by most evidence (Figure 2C).

### Earliest settlement in East Asia

The age of haplogroup O in East Asia is no more than 30 thousand years when estimated from sufficient numbers (>7) of STR markers. Therefore, haplogroup O was not the earliest Y chromosome carried by modern human into East Asia. Haplogroup C-M130 may represent one of the earliest settlements in East Asia. Haplogroup C has a high to moderate frequency in Far East and Oceania, and lower frequency in Europe and the Americas, but is absent in Africa (Figure 1). Several geographically specific subclades of haplogroup C have been identified, that is, C1-M8, C2-M38, C3-M217, C4-M347, C5-M356 and C6-P55 [25]. Haplogroup C3-M217 is the most widespread subclade, and reaches the highest frequencies among the populations of Mongolia and Siberia. Haplogroup C1-M8 is absolutely restricted to the Japanese and Ryukyuans, appearing at a low frequency of about 5% or less. Haplogroup C2-M38 is found among certain local populations on Pacific Islands from eastern Indonesia to Polynesia. Especially among the populations of Polynesia, C2 has become the modal haplogroup due to severe founder effects and genetic drift [22,26]. Haplogroup C4-M347 is the most common haplogroup among Australian aborigines, and has not been found outside of the Australian continent. Haplogroup C5-M356 has been detected with low frequency in India and the neighboring regions of Pakistan and Nepal [27,28]. C6-P55 is geographically restricted to the highlands of New Guinea (P55 has been moved to private in the latest Y chromosome tree) [29]. This wide distribution pattern of C-M130 suggests that C-M130 might have arisen somewhere in mainland Asia before modern humans arrived in Southeast Asia.

To give a clear picture about the origin and migration of haplogroup C, Zhong *et al.* typed 12 Y-SNPs and 8 Y-STRs among 465 haplogroup C individuals from 140 East and Southeast Asian populations. A general south-to-north and east-to-west decline of C3 Y-STR diversity was observed with the highest diversity in Southeast Asia, which supports a single coastal northward expansion route of haplogroup C3 in China about 32 to 42 thousand years ago using the ASD method with an average Y-STR evolutionary mutation rate of 0.00069 per locus per 25 years [25] (Figure 2A). The arrival of haplogroup C in Southeast Asia and Australia must be much earlier than that time at around 60 thousand years ago. Therefore, populations with haplogroup C must have settled in East Asia some 10 thousand years earlier than those with haplogroup O.

### Genetic legacy of the Paleolithic black Asians

**Figure 3:**
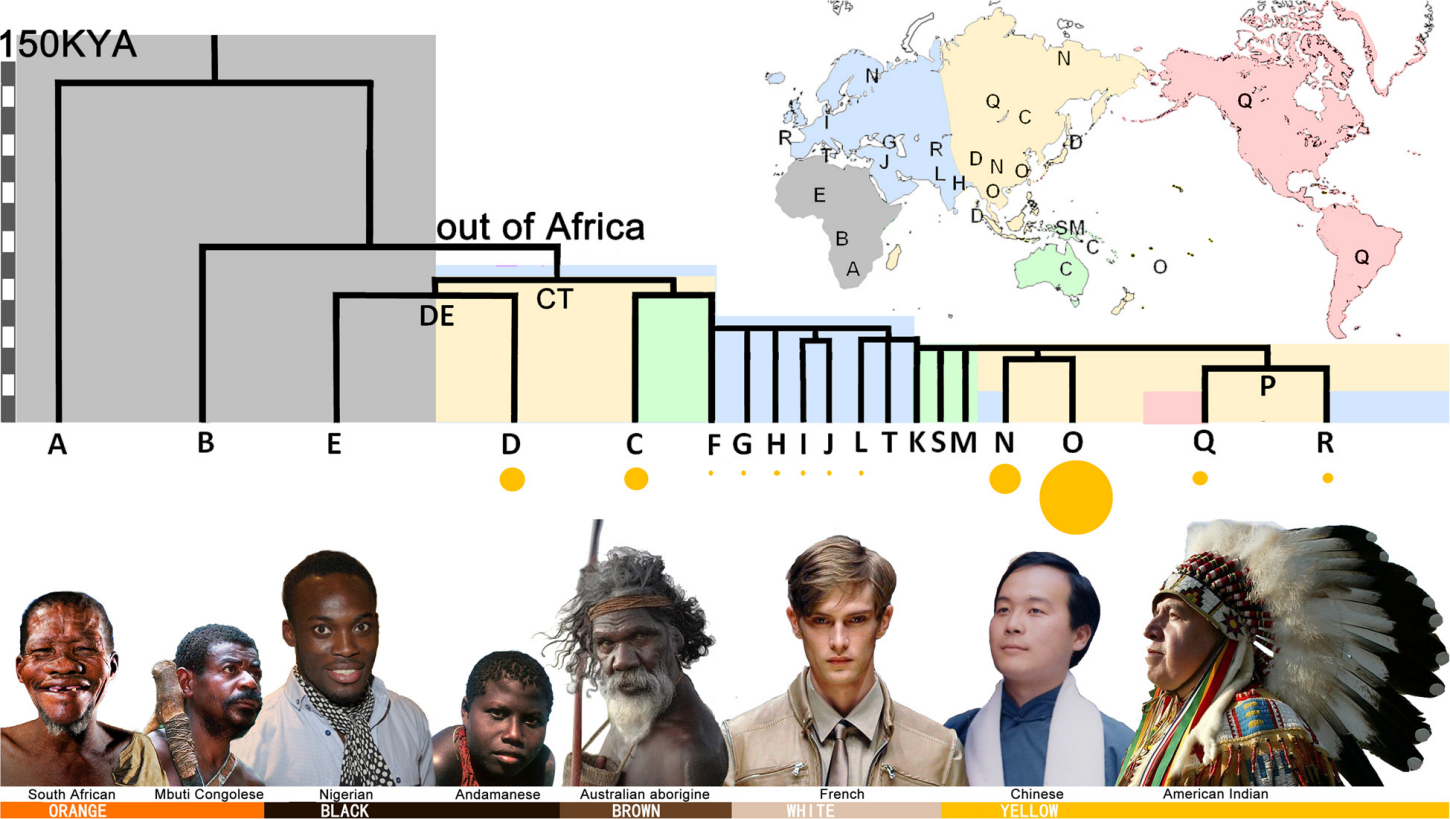
**The trunk of the Y chromosome haplogroup tree and illustrated people with the relevant haplogroups.** Background colors of the tree correspond to geographic regions marked on the world map. The sizes of the nodes below the tree correspond to the frequencies of the haplogroups in East Asia.

The migration history of haplogroup D-M174 is most mysterious. By now, we have known little about the origin and dispersal of this haplogroup. This haplogroup was derived from African haplogroup DE-M1 (YAP insertion) and is associated with a short black Asian physical style. Haplogroups E and D are brother haplogroups. While haplogroup E was carried westwards to Africa by the tall black people, haplogroup D might have been carried eastwards to East Asia by the short black people (Figure 3).

Haplogroup D-M174 has high frequencies in the Andaman Negritos, the northern Tibeto-Burman populations and the Ainu of Japan, and also appears at low frequencies in other East and Southeast Asian and Central Asian populations (Figure 1) [20,22,30,31]. A northern Tibeto-Burman population, the Baima-Dee, comprises nearly 100% of haplogroup D. There are three main subclades of haplogroup D, that is, D1-M15, D2-M55 and D3-P99, and many unclassified minor sub-haplogroups. Haplogroup D1-M15 is prevalent in the Tibetans, Tangut-Chiang and Lolo, and is also found at very low frequencies among the mainland East Asian populations [32,33]. Haplogroup D2-M55 is restricted to various populations of the Japanese Archipelago. Haplogroup D3-P99 is found at high frequencies among Tibetans and several Tibeto-Burman minorities in Sichuan and Yunnan provinces that reside in close proximity to the Tibetans, such as Pumi and Naxi [32]. The paragroup D* is restricted to the Andaman Islands [31], which has been isolated for at least 20 thousand years. Some other minor haplogroups, also included in D*, can be found around Tibet. Most of the populations with haplogroup D have very dark skin color, including the Andamanese, some of the Tibeto-Burman and Mon-Khmer people. The Ainu people may have developed pale skin to absorb more ultraviolet light in high latitude regions.

For the origin of haplogroup D, Chandrasekar *et al.* suggested that the CT-M168 gave rise to the YAP insertion and D-M174 mutation in South Asia based on their findings of the YAP insertion in northeast Indian tribes and the D-M174 in Andaman islanders [34]. In that case, haplogroup E with YAP insertion might also have an Asian origin. However, this hypothesis is seldom supported by any evidence. If haplogroup D originated in Africa, it is most mysterious how it has traveled through the populations with haplogroups CF to East Asia.

Another mystery is how haplogroup D has migrated from southwestern East Asia all the way to Japan. It could have gone either through mainland East Asia or through Sundaland (Figure 2B). The mainland route seems to be shorter than the Sundaland route. Shi *et al.* proposed that the northward expansion of D-M174 to western China might predate the migrations of other major East Asian lineages at about 60 thousand years ago using the ASD time estimation method with an average Y-STR evoltionary mutation rate of 0.00069 per locus per 25 years. Subsequently, these frontier populations could have traveled eastward through a northern route via Korea or through a southern route via Taiwan and a Ryukyu land bridge to Japan, where they might have met the earlier Australian style settlers. The current relic D-M174 in East Asia was probably edged out of eastern China by the later northward migration of haplogroup O and the Neolithic expansion of Han Chinese [32]. However, there has never been any evidence from genetics or archaeology that haplogroup D2 or Negritos have migrated to eastern China. In contrast, there are still many Negrito populations in Sundaland from Malaya to the Philippines. It was possible that Negritos occupied the whole of Sundaland in the late Paleolithic Age. Therefore, these populations might move directly from the Philippines to Taiwan and Ryukyu. The only problem is that no haplogroup D has been found in the Negritos in the Philippines. Their paternal lineages might have been replaced by the expansion of haplogroups C2 and K from Papua around 18 thousand years ago using the BATWING time estimation method [35] or a more recent migration of haplogroup O from mainland East Asia [36]. However, due to the lack of data, the history of haplogroup D, as a genetic legacy of the Paleolithic Age in East Asia, remains a mystery.

### Recent immigrations to and from the Northwest

Haplogroup O has a brother haplogroup, N-M231, which reaches the highest frequency in northern Eurasia, especially among most of the Uralic populations, including Finnic, Ugric, Samoyedic and Yukaghir people, as well as some Altaic and Eskimo populations in northern Siberia. It also appears at a low frequency in East Asia (Figure 1) [30,37]. Detailed analysis of haplogroup N suggested a more recent expansion on a counter-clockwise northern route from inner East Asia or southern Siberia about 12 to 14 thousand years ago using the ASD time estimation method with an average Y-STR evolutionary mutation rate of 0.00069 per locus per 25 years, which explains the high frequency of haplogroup N in northeast Europe [37]. Subclade N1a-M128 is found at low frequency among populations in northern China, such as Manchu, Xibe, Evenks and Korean, and also among some Turkic populations in Central Asia. Haplogroup N1b-P43 is approximately six to eight thousand years old using the ASD time estimation method with an average Y-STR evolutionary mutation rate of 0.00069 per locus per 25 years and probably originated in Siberia. N1b is prevalent in the Northern Samoyeds, and also occurs at low to moderate frequencies among some other Uralic and Altaic peoples [38,39]. The most frequent subclade N1c-Tat arose probably in China around 14 thousand years ago (the ASD method with a mutation rate of 0.00069 per locus per 25 years) and subsequently experienced a series of founder effects or strong bottlenecks in Siberia and a secondary expansion in East Europe [37]. These studies traced the origin of haplogroup N to southwestern China and Southeast Asia. Thus, it was a long march for early people with haplogroup N to take across the continent from Southeast Asia to Northern Europe.

The migration of haplogroup N is another evidence for the southern origin of the East Asians. However, there were still studies opposing the southern origin of East Asians. Karafet *et al.* examined 52 Y-SNPs in 1,383 individuals of 25 populations from East Asia and Central Asia. They found the average pairwise difference among haplogroups was noticeably smaller in southern East Asia and there was no genetic divergence between southern and northern East Asia [30]. Xue *et al.* applied a Bayesian full-likelihood analysis to 45 Y-SNPs and 16 Y-STRs data from 988 men of 27 populations from China, Mongolia, Korea and Japan. They reported the Y-STRs have a higher diversity in northern East Asian populations than that in southern populations. The northern populations expanded earlier than the southern populations [40]. However, Shi *et al.* pointed out that the larger diversity among Y-chromosome haplogroups observed in northern East Asia claimed by Karafet *et al.* is actually a false impression due to the recent population admixture. The study of Xue *et al.* has a similar drawback. The large gene diversity observed in Mongols, Uighurs and Manchurians was probably due to their recent extensive admixture with Central Asian, West Eurasian and Han Chinese populations. Furthermore, the southern populations studied by Xue *et al.*were not sufficient and the within-population bottleneck effect caused by long-time geographic isolation might have a great impact on gene diversity estimation [32].

The subsequent debate focuses on how to interpret the Central Asia- and West Eurasia-related genetic components in East Asia. Zhong *et al.* sampled 3,826 males from 117 populations and performed high-resolution genotyping to address this problem. In the study by Zhong *et al.*, haplogroups O-M175, C-M130, D-M174 and N-M231 still suggest the substantial contribution of the southern route. However, the Central Asia- and West Eurasia-related haplogroups, such as haplogroups R-M207 and Q-M242, occur primarily in northwestern East Asia and their frequencies gradually decrease from west to east. In addition, the Y-STR diversities of haplogroups R-M207 and Q-M242 also indicate the existence of northern route migration about 18,000 years ago (ASD method with a mutation rate of 0.00069 per locus per 25 years) from Central Asia to North Asia, and a recent population admixture along the Silk Road since about 3,000 years ago [16].

### Mother language or father language

The genetic patterns in human societies are often influenced by their cultural practices, such as residence patterns and subsistence strategies. Y chromosomes of East Asian populations have played an important role in documenting such influences; for example, relationships among patrilocal populations should have stronger associations with Y chromosomes than with mtDNA. East Asian languages show strong association with paternal lineages of Y chromosomes [18,23,25,41,42] and whole genomic diversity [43], but not maternal lineages of mtDNA. The Y chromosome haplogroup O3-M134 is associated with Sino-Tibetan speakers [18,41]; O2-M95 is associated with Austro-Asiatic speakers [42]. Moreover, phylogenetic structure among the linguistic families is also supported by Y chromosomes but not by whole genomic diversity; for example, linguistic affinity between Hmong-Mien and Austro-Asiatic languages was proved by Y chromosome marker O3-M7 [23], and that between Tai-Kadai and Austronesian languages by O1-M119 [44].

Another interesting topic is about language expansion and Y chromosome distribution patterns, That is, whether modern languages underwent a serial founder effect during their expansion. Atkinson reported a decreased trend of phonemic diversity (vowel qualities, tones and consonants) from Africa that indicated the African exodus of modern languages [45]. However, this hypothesis is not widely accepted among linguists. Wang *et al.* argued that Atkinson’s claim was only supported when the phonemic diversities were divided into three or five levels. Analyses using raw data without simplification suggest a decline from central Asia rather than from Africa [46], although this result might not be the last word on language origin and expansion [47]. The distribution pattern of the phonemic diversity may not reflect the initial origin of modern humans, but probably indicates the secondary expansion of modern humans in south-central Asia. The Asian expansion is also supported by Y chromosome evidence. The Y chromosome haplogroup phylogenetic tree has an African root, but only haplogroups A and B were African aboriginal types, that is, these two haplogroups have probably never left Africa. All the other haplogroups, all within CF and DE, derived from one ancient type with a marker M168 out of Africa around 50 to 70 thousand years ago. They might have expanded from western Asia around 30 to 40 thousand years ago, and given birth to all the haplogroups from C to T [3]. Therefore, the haplogroup with highest frequency in Africa, haplogroup E, was probably also from the Asian Expansion. In Africa, Haplogroup A is mostly in Khoisan and Saharan populations, and haplogroup B is mostly in Pygmies and other populations around Congo. Y lineages of the major populations of Africa, sometimes called Bantu or Niger-Congo people, were then most probably returned from Asia. The back migration of DE-M1 (YAP insertion) from Asia to Africa have already been proposed by Altheide and Hammer [48] and Hammer *et al.*[49,50]; however, this has been questioned by Underhill *et al.*[51] and Underhill and Roseman [52]. Nevertheless, the high frequencies of haplogroup R1-M173 in Cameroon also supported the back migration from Asia to sub-Saharan Africa [53].

The association between languages and Y chromosomes, but not mtDNA, might reflect sex-biased migrations due to patrilocality. Patrilocality refers to the social system in which a married couple resides with or near the husband's parents. Forster *et al.* suggested that it may often be the language of the father that is dominant within the family group if the parents have different linguistic backgrounds [54]. However, as the whole genomic diversity is also associated with linguistic families, both paternal and maternal lineages must have been well kept since the linguistic families emerged. Therefore, the loss of association between mtDNA and languages might not simply be explained by a social nature of adopting women. There might be a higher effective population size in ancient populations for females than for males due to frequent hunting activities and wars, and thus, the original mtDNA variation of a language group was less affected by genetic drift. Other interpretations might also be possible, such as preferential males (discussed below), the number of offspring, the different mutation rates, and so on.

Other cultural practices, such as agriculture, military affairs or social prestige, can also have impacts on genetic patterns. The spread of culture is explained by two alternative demographic scenarios: the demic diffusion model, which involves mass movement of people; and the cultural diffusion model, which refers to the movement of ideas and practices rather than people and involves limited genetic exchange between people [55]. For example, Wen *et al.* analyzed the genetic pattern of Y-chromosomes and mtDNA variation in 28 Han Chinese populations. According to their data, northern and southern Hans share similar Y chromosome haplogroup frequencies but exhibit more differences in their mtDNA lineages. The substantial movements of the northern immigrants fleeing from warfare and famine have led to the transition in genetic structure in southern China. They concluded that the pattern of the demographic expansion of Han people and the southward diffusion of Han culture was consistent with the demic diffusion model, and males played a bigger role than females in this expansion [41].

### Y chromosomes of eminent persons in history

Population expansion can also be associated with specific social prestige, for example, the increased reproductive fitness as in Genghis Khan’s case. Genghis Khan (1162 to 1227) established the largest contiguous empire in history. He and his patrilineal relatives had numerous offspring due to their high social status, which led to the increase in frequency of their Y lineage. As a result, his Y chromosome (C3*xC3c, star-cluster) is widespread from the Pacific to the Caspian Sea and makes up an estimated 0.5% of the world’s male population today [56]. Interestingly, the Kereys tribe in Kazakhstan reaches the highest frequency (76.5%) of the C3* star-cluster Y-chromosomes [57]. It is unlikely that C3* star-cluster in Kereys was attributed to Genghis Khan, doubting the origin of C3* in Genghis Khan’s family. Nonetheless, social selection plays an important role in the expansion of this C3* star-cluster. Similarly, Y chromosome haplogroup C3c-M48 was suggested to be spread by the Qing Dynasty (1644 to 1912) Manchurian nobility, comprising about 3.3% of the males sampled from East Asia [58].

Similar to the study of Genghis Khan’s lineage, Y chromosome can be used to study the eminent persons in history. Most people now inherit surnames from their fathers; and similarly, most men also inherit the Y chromosomes from their fathers. Therefore, men sharing the same surname are expected to have similar Y chromosomes. Combined with the study of stemma records, Y chromosome study can reveal the relationships of individuals in ancient history [59]. Wang *et al.* collected some present clans with full records of 70 to 100 generations claimed to be descendants of the famous Chinese Emperor CaoCao or Marquis Cao Can, and validated the records by comparing the Y chromosomes. The Y chromosome haplotype of the descendants of Emperor CaoCao (O2-M268) is different from that of the Marquis Cao Can (O3-002611). Therefore, Cao Cao’s claim of aristocratic ancestry from Cao Can, which has been doubted for almost 1,800 years is, therefore, not supported by genetic evidence [60]. Ancient DNA extracted from a tooth of Cao Cao’s grand-uncle also supported the conclusion drawn from the present Y chromosomes that the haplogroup of Emperor Cao was O2*-M268. Emperor Cao’s father was most probably adopted from his grandfather’s own clan rather than from beggardom [61].

The application of genetics to the studies of the ancient history will continue to grow. In genealogy, for example, it would be useful to investigate the genetic links among clans with the same surname to establish the missing records of family stemma [62]. The deep-rootedpedigrees are also of great value for studying the Y-chromosome evolution. Xue *et al.* sequenced the Y chromosomes of two individuals separated by 13 generations and estimated the human Y chromosome base-substitution mutation rate as 3.0×10^-8^ mutations/nucleotide/generation [63]. Deeper pedigrees will be better materials to make a more accurate estimation.

## Conclusions

The Y chromosome plays an important role in unraveling the entangled history of modern human populations in East Asia. Although many questions remain unresolved, a clear framework of the prehistory has been obtained. Four Y chromosome haplogroups C, D, O and N, accounted for more than 90% of the East Asian Y chromosomes, are suggested to have Southeast Asian origins, carried by three waves of migrations. The distributions of western Eurasia specific Y chromosome haplogroups E, G, H, I, J, L, Q, R and T in northwest China reflect the recent gene flows from the west and the probable northern route migration. A west-to-east decline of these western haplogroups was clearly observed.

However, current Y chromosome research in East Asia is limited in two important aspects. The first limit is the poor resolution for those East Asian specific Y chromosome branches, such as haplogroup O-M175. Despite the huge population of haplogroup O, there have been fewer markers defined in haplogroup O than in haplogroups R and E. For instance, three Y-SNP markers, 002611, M134 and M117, represent about 260 million people in East Asia, but downstream markers are far from enough to reveal informative genetic substructures of those populations. The second limit is inaccurate estimation of lineage and population divergence time as mentioned at the beginning.

The advent of next-generation sequencing technology made it possible to sequence the entire Y chromosome in numerous human individuals and in deep-rooting pedigrees. For instance, the 1000 Genomes Project Consortium has already sequenced the Y chromosomes at an average depth of 1.83 in 77 males in the low-coverage project, and 15.23 in the two trio fathers [64]. Further deep sequencing will offer a solution for both enhanced Y chromosome phylogenetic resolution and accurate calibration of the molecular clock in evolutionary studies.

## Abbreviations

ASD: Average squared difference; Bp: Base pairs; LGM: Last Glacial Maximum; mtDNA: Mitochondrial DNA; NRY: Non-recombining portion of the Y chromosome; SNP: Single nucleotide polymorphism; STR: Short tandem repeat.

## Competing interests

The authors declare that they have no competing interests.

## Authors’ contributions

CCW and HL wrote the manuscript and performed graphical work. Both the authors read and approved the final manuscript.

## Supplementary Material

Additional file 1 Phylogenetic trees of Y chromosomal haplogroup O-M175, C-M130, D-M174, and N-M231. Click here for file
