# Supplementary figures and images for: Inferring human history in East Asia from Y chromosomes
Source: Investig Genet. 2013 Jun 3;4:11. doi: 10.1186/2041-2223-4-11 (PMC3687582; doi:10.1186/2041-2223-4-11)

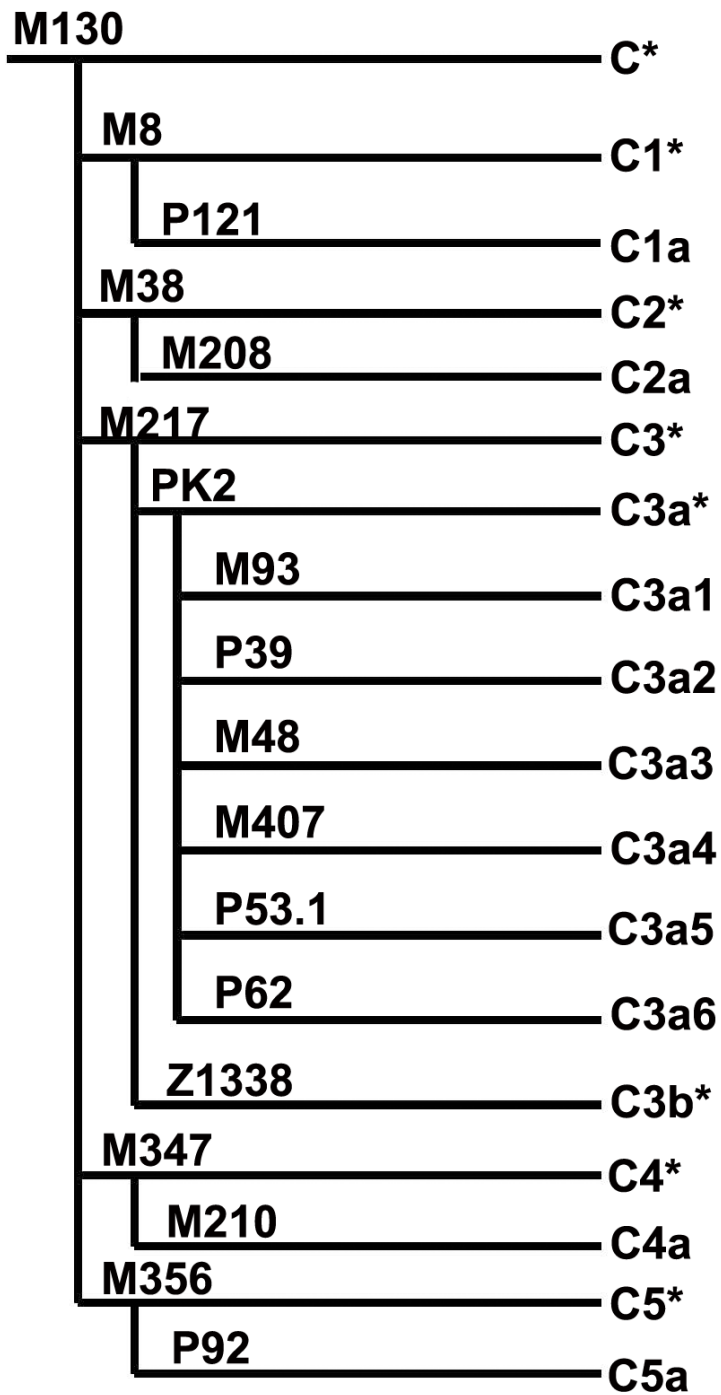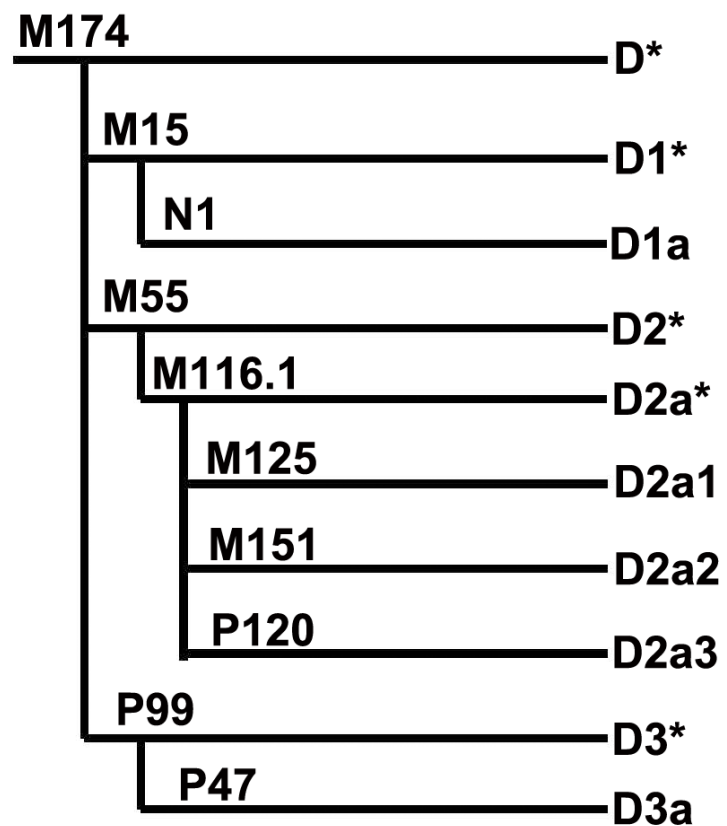

Supplement: Additional file 1 — Phylogenetic trees of Y chromosomal haplogroup O-M175, C-M130, D-M174, and N-M231. [file 2041-2223-4-11-S1.pdf]
